# Microfluidic electrochemical cell for *in situ* structural characterization of amorphous thin-film catalysts using high-energy X-ray scattering

**DOI:** 10.1107/S1600577519007240

**Published:** 2019-08-09

**Authors:** Gihan Kwon, Yeong-Ho Cho, Ki-Bum Kim, Jonathan D. Emery, In Soo Kim, Xiaoyi Zhang, Alex B. F. Martinson, Davd M. Tiede

**Affiliations:** a Argonne Northwestern Solar Energy Research (ANSER) Center, Northwestern University, 2145 Sheridan Road, Tech Room L110, Evanston, IL 60208-3113, USA; b Northwestern-Argonne Institute of Science and Engineering, Northwestern University, 2205 Tech Drive, Evanston, IL 60208, USA; c Materials Science Division, Argonne National Laboratory, 9700 South Cass Ave, Lemont, IL 60439, USA; d Chemical Sciences and Engineering Division, Argonne National Laboratory, 9700 South Cass Ave, Lemont, IL 60439, USA; e Nano Fabrication Laboratory, Research Institute of Advanced Materials, Department of Materials Science and Engineering, Seoul National University, 599 Gwanak-ro, Gwanak-gu 151-744, South Korea; f X-ray Science Division, Argonne National Laboratory, 9700 South Cass Ave, Lemont, IL 60439, USA

**Keywords:** atomic layer deposition, electrode architectures, pair distribution functions, high-energy X-ray scattering, catalysts, electrochemistry, ultra-thin films

## Abstract

Porous, high-surface-area electrode architectures are described that allow structural characterization of interfacial amorphous thin films with high spatial resolution under device-relevant functional electrochemical conditions using high-energy X-ray (>50 keV) scattering and pair distribution function (PDF) analysis. Porous electrodes were fabricated from glass-capillary array membranes coated with conformal transparent conductive oxide layers, consisting of either a 40 nm–50 nm crystalline indium tin oxide or a 100 nm–150 nm-thick amorphous indium zinc oxide deposited by atomic layer deposition. These porous electrodes solve the problem of insufficient interaction volumes for catalyst thin films in two-dimensional working electrode designs and provide sufficiently low scattering backgrounds to enable high-resolution signal collection from interfacial thin-film catalysts. For example, PDF measurements were readily obtained with 0.2 Å spatial resolution for amorphous cobalt oxide films with thicknesses down to 60 nm when deposited on a porous electrode with 40 µm-diameter pores. This level of resolution resolves the cobaltate domain size and structure, the presence of defect sites assigned to the domain edges, and the changes in fine structure upon redox state change that are relevant to quantitative structure–function modeling. The results suggest the opportunity to leverage the porous, electrode architectures for PDF analysis of nanometre-scale surface-supported molecular catalysts. In addition, a compact 3D-printed electrochemical cell in a three-electrode configuration is described which is designed to allow for simultaneous X-ray transmission and electrolyte flow through the porous working electrode.

## Abstract 2

Porous, high-surface-area electrode architectures are described that allow structural characterization of interfacial ultra-thin catalyst films under device-relevant functional electrochemical conditions using high-energy X-ray (>50 keV) scattering and pair distribution function analysis.

## 1. Introduction   

*In situ* structural characterization of ultra-thin electrochemically active materials with atomic scale resolution is required to achieve an understanding of the reaction mechanisms and for the design of new materials. The resolution of structure during electrochemical and catalytic function is important for a range of electrochemical energy storage (Wiaderek *et al.*, 2013 ▸), electrocatalysis (Danilovic *et al.*, 2014 ▸; Ocko *et al.*, 1990 ▸; Tidswell *et al.*, 1993 ▸; Ingham *et al.*, 2008 ▸) and solar photoelectrochemical (Reece *et al.*, 2011 ▸; Nocera, 2012 ▸) applications.

The extensive literature describes electrochemical cell designs for *in situ* characterization of interfacial electrochemistry using a variety of X-ray techniques, including X-ray absorption fine-structure (XAFS) spectroscopy (Merte *et al.*, 2012 ▸; Virtanen, 2002 ▸; Watanabe *et al.*, 2007 ▸; Kordesch & Hoffman, 1984 ▸), X-ray reflectivity, standing wave, diffraction (Veder *et al.*, 2011 ▸; Wang *et al.*, 1992 ▸; Tidswell *et al.*, 1993 ▸; Ingham *et al.*, 2008 ▸; Renner *et al.*, 2007 ▸; Robinson & O’Grady, 1993 ▸; Koop *et al.*, 1998 ▸; Morcrette *et al.*, 2002 ▸; Tamura *et al.*, 2002 ▸) and high-energy X-ray scattering (HEXS) (Borkiewicz *et al.*, 2012 ▸; Jensen *et al.*, 2015 ▸). XAFS data collection in the fluorescence mode has proven to be a sensitive and well suited technique for interrogating metal-atom oxidation states and the electronic and coordination structures of interfacial thin-film catalysts (Risch *et al.*, 2015 ▸, 2012 ▸; Kanan *et al.*, 2010 ▸). XAFS provides an accurate measure of metal-to-ligand atom distances within the inner-coordination shell. However, information on atom pair distances with outer-shell coordination (4 Å–10 Å) and longer-range structures are typically limited in XAFS measurements for disordered, amorphous, molecular and non-crystalline materials (Du *et al.*, 2012 ▸; Blakemore *et al.*, 2013 ▸; Mulfort *et al.*, 2013 ▸; Yang *et al.*, 2016 ▸). X-ray scattering techniques provide a complementary measure of the outer shell and longer-range atomic order across the 1 Å to 100 nm distance range (Mulfort *et al.*, 2013 ▸; Blakemore *et al.*, 2013 ▸). Grazing-incidence X-ray scattering techniques are widely employed for interfacial thin-film characterization, but are typically implemented with X-ray energies of less than 30 keV, which limits spatial resolution to 0.1 nm because of the shorter momentum transfer (*q*).

HEXS and atomic pair distribution function (PDF) techniques are well suited for structural characterization of functional materials with sub-angstrom spatial resolution (Billinge & Kanatzidis, 2004 ▸; Juhás *et al.*, 2006 ▸; Billinge & Levin, 2007 ▸; Michel *et al.*, 2007 ▸; Chupas *et al.*, 2009 ▸). PDF measurements achieve spatial resolution comparable to XAFS, and the two techniques complement each other to provide high-resolution structure characterization across the full-length scale from inner-shell metal coordination to intermediate-range distances (Németh *et al.*, 2012 ▸; Mulfort *et al.*, 2013 ▸; Blakemore *et al.*, 2013 ▸; Yang *et al.*, 2016 ▸). However, a challenge in applying PDF methods with high spatial resolution for the analysis of ultra-thin films arises from the reduced atomic scattering cross-sections for HEXS and the small interaction volume within ultra-thin films which limit the amplitude of scattering signals. PDF techniques have been developed that allow *operando* measurements of the structures of micrometre-scale electrode materials during electrochemical cycling in battery devices (Jung *et al.*, 2015 ▸; Borkiewicz *et al.*, 2012 ▸, 2015 ▸; Wiaderek *et al.*, 2013 ▸). The use of high-flux synchrotron sources for HEXS and PDF analysis of sub-micrometre films on planar glass supports has been demonstrated through the resolution of the PDF for amorphous thin films of FeSb_3_ having a thickness of 360 nm (Jensen *et al.*, 2015 ▸), although the thin glass support did not allow for electrochemical addressing of the supported film (Jensen *et al.*, 2015 ▸). Furthermore, the sensitivity of HEXS to FeSb_3_ thin films is enhanced by more than a factor of ten compared with comparable oxide films. With the development of micro-focusing optics for high-energy X-rays, recent work has demonstrated high-resolution PDF measurements for surface-supported films of ZrO_2_, HfO_2_ (Dippel *et al.*, 2019 ▸) and Pt (Roelsgaard *et al.*, 2019 ▸) supported films that are a few nanometres thick using grazing-incidence HEXS.

In this report, we describe an alternate strategy for PDF analysis of thin-film transition-metal oxides by introducing a 3D porous electrode architecture. A larger interaction volume enables interfacial thin films of first-row transition-metal amorphous oxides and other non-crystalline materials with relatively low-*Z* constituent atoms to be characterized by HEXS, and without the requirement for a micro-focused X-ray beam. We study amorphous cobalt-containing thin films formed by anodic electrochemical deposition from dilute cobalt nitrate solutions in the presence of phosphate or borate as the electrolyte anion (Kanan & Nocera, 2008 ▸; Surendranath *et al.*, 2009 ▸). The corresponding amorphous CoO_*x*_-Pi and CoO_*x*_-Bi thin films have been widely investigated as water-oxidizing catalysts for photoelectrochemical device (artificial leaf) applications (Nocera, 2012 ▸; Liu *et al.*, 2016 ▸; González-Flores *et al.*, 2015 ▸; Risch *et al.*, 2015 ▸). The amorphous domain for CoO_*x*_-Pi and CoO_*x*_-Bi share a common edge-sharing *O*_*h*_ CoO_6_ cobaltate core (Farrow *et al.*, 2013 ▸; Du *et al.*, 2012 ▸; Kwon *et al.*, 2015 ▸; Kanan *et al.*, 2010 ▸; Risch *et al.*, 2015 ▸, 2012 ▸) but differ in domain size. CoO_*x*_-Pi consists of small monolayer cobaltate domains, with ∼11 Å maximum dimension (Du *et al.*, 2012 ▸; Farrow *et al.*, 2013 ▸), while CoO_*x*_-Bi shows larger, ∼20 Å, domains, with turbostratic disordered stacking of approximately three layers (Farrow *et al.*, 2013 ▸; Kwon *et al.*, 2015 ▸). CoO_*x*_-Pi and CoO_*x*_-Bi serve as useful reference materials for developing capabilities for PDF characterization of electrode-supported thin-film disordered and molecular-dimensioned materials.

The described 3D porous working electrode (WE) improves the intensity of the scattered signal for thin films compared with a 2D planar WE design by a factor that scales with surface area. We demonstrate an electrode design consisting of a glass-capillary array (GCA) that is selectable with pore diameters in the 2 µm to 100 µm range, and is conformally coated with a transparent conductive oxide (TCO) using atomic layer deposition (ALD). This approach provides porous electrode architectures with surface chemistries that can be tuned to match those utilized in a range of electrochemical (Danilovic *et al.*, 2014 ▸; Ocko *et al.*, 1990 ▸; Tidswell *et al.*, 1993 ▸; Ingham *et al.*, 2008 ▸) and solar photoelectrochemical (Reece *et al.*, 2011 ▸; Nocera, 2012 ▸) devices. With electrodes fabricated from GCAs with 40 µm-diameter pores and coated with either a 40 nm indium tin oxide (ITO) or a 50 nm–100 nm amorphous indium zinc oxide (a-IZO) layer (Lee *et al.*, 2014 ▸), PDFs were readily obtained with 0.2 Å spatial resolution for amorphous cobalt oxide water-oxidation catalyst films having thicknesses down to 60 nm. This level of spatial resolution resolves the cobaltate domain size and structure, the presence of defect sites assigned to the domain edges (Kwon *et al.*, 2015 ▸; Du *et al.*, 2012 ▸) and the change in fine structure associated with redox state cycling, thereby enabling quantitative structure–function understanding. High-resolution PDF measurements on films with thicknesses below 60 nm are also accessible by use of higher-surface-area substrates. The results suggest the opportunity to perform PDF structural analyses on monolayers of surface-bound molecular complexes by extending porous electrode designs to sub-micrometre scale pores.

## 2. Experimental procedures   

### 2.1. Electrode design and preparation   

We investigated a number of planar and 3D porous electrode architectures as WEs for *in situ* interrogation of amorphous thin films of first-row transition-metal oxides using HEXS measurements under controlled electrochemical conditions. Planar electrodes were ITO-coated, 180 nm thick, ≤10 Ω per square, on polished glass and precision cut to 3 mm × 10 mm × 1.1 mm (Präzisions Glas & Optik GmbH). A 3D electrode design that we found to be amenable to a broad range of electrochemical applications used a GCA plate wrapped with a TCO layer by ALD (Elam, Baker, Martinson *et al.*, 2008 ▸; Lee *et al.*, 2014 ▸).

**Figure 1:**
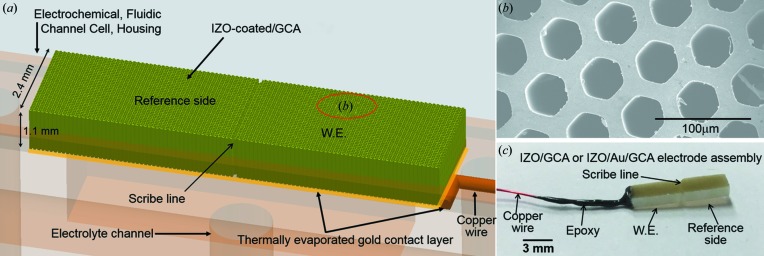
GCA electrode assembly. (*a*) Schematic diagram of the microporous electrode assembly, composed of a 50 nm-thick IZO-coated 2.4 mm × 1.1 mm × 10 mm GCA with 40 µm pores, IZO/Au/GCA. The bottom surface was further coated with a thermally evaporated Au (100 nm) layer that provided an efficient electrical contact. The scribe line shown in parts (*a*) and (*c*) separates the electrical connection between the WE and scattering reference sides. (*b*) Picture of the 40 mm porous IZO/GCA. (*c*) Image of the experimental IZO/Au/GCA electrode assembly, viewed from the side with the electrical-contacting copper wire. The epoxy seal of the contact region shown in (*c*) is not illustrated in (*a*) for better visualization.

An illustration of the TCO-coated GCA electrode assembly, TCO/GCA, is shown in Fig. 1 ▸. The borosilicate GCA has a 1.1 mm thickness with 40 µm pores arrayed in a hexagonal honeycomb structure (58% pore density), was purchased from InCom Inc, and cut into 2.4 mm × 10 mm size. Conformal TCO coatings were produced with a commercial wafer-style reactor (Savannah 200, Cambridge Nanotech) at 100°C under low vacuum in flowing N_2_. In one version, a 32 nm layer of ITO was deposited as a crystalline conductive oxide by ALD in a process adapted from previous reports (Elam, Baker, Martinson *et al.*, 2008 ▸; Elam *et al.*, 2006 ▸; Elam, Baker, Hryn *et al.*, 2008 ▸). Briefly, 20 super cycles of 10 cycles each (200 ALD cycles total) were run, with each super-cycle-dosing schedule consisting of: {[cyclopentadienyl­indium(I)-soak]–purge–ozone–purge}_8_ + {[tetrakis­(dimethyl­amido)tin(IV)-soak]–purge–ozone–purge} with timing [(1–30)–30–60–25]_8_–[(1–15)–30–60–25] in seconds at a temperature of 225°C with In and Sn precursors heated to 70°C and 55°C, respectively. The relatively long 30 and 15 s soak times are performed without pumping during and shortly after each metal organic precursor dose to ensure complete diffusion of the ALD precursors into the high-aspect-ratio substrates. In a second version, ALD of a 50 nm–100 nm IZO was used to provide an amorphous conductive oxide layer with a conductivity comparable to ITO. The ALD process was adapted from a previous report (Lee *et al.*, 2014 ▸). Briefly, 200 super cycles of 10 (1 Zn, 9 In) cycles each (720 ALD cycles total) were run, with each super-cycle-dosing schedule consisting of: [(trimethylindium-soak)–purge–water–purge]_9_ + [(diethylzinc-soak)–purge–ozone–purge] with timing [(1–5)–20–3–20]_9_–[(1–5)–20–0.5–20] in seconds at a temperature of 220°C. The relatively long five-second soak time is performed without pumping during and shortly after each metal organic precursor dose to ensure complete diffusion of the ALD precursor species, specifically, the In precursor on the ZnO_*x*_ surface and the Zn precursor on the InO_*x*_ surface during the ALD reaction cycles. Finally, the ITO- and IZO-coated porous electrodes were annealed at 400°C under flowing N_2_ for 1 h to improve the conductivity. An ∼80% ratio of indium to zinc in a-IZO films shows a low resistivity of 3.9 × 10^−4^ Ω cm and high electron mobility, >50 cm^2^ V^−1^ s^−1^, one of the highest among the reported ALD-grown transparent conducting oxides, while still avoiding crystallinity (Lee *et al.*, 2014 ▸).

For both versions of the TCO/GCA electrode assembly, a gold thin film was deposited by thermal evaporation on one surface of the GCA prior to TCO deposition in order to reduce the sheet resistance (resistance to electrical conduction from the wire contact along the face of the membrane). A capillary-cutting stone (HR4-334, Hampton Research) was used to circumscribe a ‘scratch’ through the conductive layers, creating electrochemically active and inactive zones. The inactive side provided a scattering background for the TCO/GCA electrode assemblies during *operando* electrochemical measurements. Figs. 1 ▸(*b*) and 1 ▸(*c*) show scanning electron and optical micrographs, respectively, of the 1.1 mm × 2.4 mm × 10 mm (height × width × length) electrode assembly.

### 2.2. Beamline electrochemical cell   

**Figure 2:**
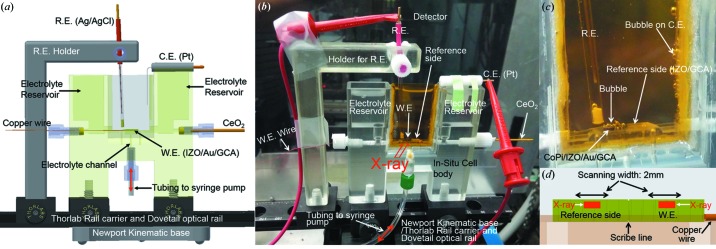
(*a*) CAD of the *in situ* electrochemical cell. (*b*) A photograph of the *in situ* electrochemical cell assembly installed during HEXS data acquisition and consisting of electrolyte reservoir, *in situ* cell body with syringe pump, WE, RE, CE, holder for RE, and mounting stage including Newport Kinematic base, Thorlab rail carrier and dovetail optical rail. Polyimide film (yellow) holds electrolyte. (*c*) Magnification of the WE and reference side during electrolysis after 1 h of electrolysis. (*d*) Scaled, schematic side-view drawing showing the horizontal scanning width of X-ray on IZO/Au/GCA electrode. The size of the incoming X-ray beam is 500 mm (width) by 300 mm (height) as indicated by the red rectangular box in (*d*).

Fig. 2 ▸(*a*) shows a computer-aided design (CAD) of the *in situ* electrochemical module holding a microporous WE used for HEXS measurements. The custom 3D-printed electrochemical cell includes an electrolyte reservoir, and the electrochemical compartment, housing the porous WE, a reference electrode (RE) and a counter electrode (CE). The cell connects to tubing which forces electrolyte flow through the porous electrode via a syringe pump. The reusable cell was fabricated with a 3D printer (Objet30 Pro, Stratasys Ltd) using optically transparent VeroClear-RGD810 (Stratasys Ltd) that permits visible inspection of the inside of the cell during electrolysis or X-ray scattering. Prior to assembly, the printed *in situ* cell was sonicated in a solution of 0.5 *M* NaOH solution in deionized water and then rinsed using deionized water. This process was repeated several times to remove low-cross-linked support materials on the surface of the printed cell.

The WE was connected with polyurethane/nylon insulated copper wire (8056, BELDEN). Silver paste (#12642-14, Electron Microscopy Science) was used to contact the IZO-coated/Au/GCA WE to the copper wire and the assembly was dried in an oven. Chemical-resistant epoxy (9340, Loctite) was applied to cover the silver paste and exposed copper wire. A silver/silver chloride (Ag/AgCl) RE was constructed from a glass tube [VitroCom, #CV2024, 2.0 mm inner diameter (ID) × 2.4 mm OD (outer diameter)], a porous Teflon frit (CH Instruments Inc., product ID: CHI_RETIP), and silver-chloride film deposition on silver wire (CHE112, CH Instruments Inc.) performed at 0.5 V versus normal hydrogen electrode (NHE) in 0.1 *M* hydrochloric acid solution for 30 min. The RE was positioned adjacent to the WE, as shown in Fig. 2 ▸(*b*). A Pt wire, shown in Fig. 2 ▸(*a*), was used as a CE. A 25 µm-thick polyimide film (Kapton, Lebow Company) shown in yellow in Figs. 2 ▸(*b*) and 2 ▸(*c*) serves as an X-ray transmissive window and is sealed with chemical-resistant epoxy (LOCTITE 9340, McMaster-Carr). The *in situ* cell was mounted on a kinematic base (BKL-4, Newport), a dovetail optical rail (RLA0600, THORLABS) and a rail carrier (RC1, THORLABS). A syringe was primed to remove bubbles through the entire cell and tubing. The syringe pump was then mounted on a programmable bidirectional syringe pump (NE-1010, New Era Pump Systems Inc.), which was connected to the electrochemical cell body through phthalate-free TYGON tubing (Saint-Gobain SE-200, 1/8 inch OD, Fisher Scientific) in a position that provided a continuous electrolyte flow through the porous WE by a pre-programmed schedule. Flowing electrolyte through the porous electrode helped to clear oxygen bubbles formed on the WE during oxygen-evolving catalysis as well as replenishing electrolyte in the pores of the WE.

### 2.3. Solution preparation and film growth   

Detailed information about the preparation of the electrolyte and electrochemical deposition of amorphous cobalt oxide water-splitting catalysts films in the presence of borate (CoO_*x*_-Bi) and phosphate (CoO_*x*_-Pi) has been previously reported (Kanan & Nocera, 2008 ▸; Risch *et al.*, 2012 ▸; Du *et al.*, 2012 ▸). For CoO_x_-Bi, thin films were deposited onto the planar ITO glass or microporous IZO-coated GCA from electrolyte solutions containing 0.1 *M* borate, pH 9.0, and 0.5 m*M* Co(NO_3_)_2_·6H_2_O (Strem Chemical Inc.), as described previously (Kwon *et al.*, 2015 ▸). A potentiostat (Epsilon, Bioanalytical Systems Inc.) was used to perform all of the three-electrode experiments. *I* (current) × *R* (resistance) compensation was not included in any of the electrochemical measurements. For CoO_*x*_-Pi catalyst deposition, electrolyte solutions of 0.1 *M* potassium phosphate (KPi, pH 7.0) containing 0.5 m*M* Co(NO_3_)_2_·6H_2_O (93-2746, Strem Chemical Inc.) were prepared from potassium phosphate (KPi, KH_2_PO_4_, P5655 at Sigma-Aldrich) with ultra-pure water (18.2 MΩ cm, MilliQ). The pH of the electrolyte solution was adjusted using concentrated potassium hydroxide (484016, Sigma-Aldrich) solution. Electrodeposition of CoO_*x*_-Pi film on planar ITO glass for the PDF measurements of CoO_*x*_-Pi by grazing incidence in the supported film and from *ex situ* powders were performed at 1.34 V versus NHE. Measurements of electrochemical potential-dependent PDF fine-structure changes were performed after a thick CoO_*x*_-Pi film was formed (1 h at 1.34 V versus NHE) and then three sets of HEXS data with 5 min signal acquisitions at 0.5 V and 1.34 V versus NHE were obtained and finally averaged for PDF analysis. The experiment is described further below. For powder X-ray scattering measurements, CoO_*x*_-Pi and CoO_*x*_-Bi were scraped off 25 cm^2^ planar ITO electrode surfaces, collected as powders and placed in 0.7 mm borosilicate glass-capillary tubing (Friedrich & Dimmock Inc.) for HEXS measurements.

### 2.4. X-ray data acquisition and processing   

HEXS 2D scattering images, using 58.7 keV (11ID-B, λ = 0.2114 Å) and 100.3 keV (6ID-D, λ = 0.1236 Å) X-ray energies, were acquired at the Advanced Photon Source (APS) at the Argonne National Laboratory using a Perkin–Elmer amorphous silicon detector (Chupas *et al.*, 2003 ▸, 2007 ▸). Sample-to-detector distances of 19 cm and 34 cm were used for the 58.7 keV (beamline 11-ID-D) and 100.3 keV (beamline 6-ID-D) measurements, respectively, and both were calibrated using a CeO_2_ powder pattern. The maximum *q* value accessible in these measurements was *q*_max_ = 4πsin(2θ/2)/λ = 24 Å^−1^ at 58.7 keV, where 2θ is the scattering angle and λ is the X-ray wavelength. 2D X-ray scattering patterns were integrated to a 1D spectrum using *Fit2D* software (Hammersley *et al.*, 1996 ▸). X-ray data were acquired using a 300 µm height × 500 µm width beam profile and with 2 mm horizontal translation of the electrochemical cell as shown in Fig. 2 ▸(*d*). The movement of the electrochemical cell during the X-ray data acquisition was used to provide spatial averaging. Both electrochemically active and inactive areas of the TCO/GCA were interrogated during the time course of the electrochemical experiments, with the latter serving as the HEXS background. The X-ray interrogated areas are illustrated by the side-view diagram in Fig. 2 ▸(*d*). Background-scattering patterns of electrolyte/WE were subtracted from the sample scattering patterns of catalyst/electrolyte/WE, and corrected for X-ray polarization, sample absorption and Compton scattering using the *PDFgetX2* program (Qiu *et al.*, 2004 ▸). The details of background subtraction have been previously described (Du *et al.*, 2012 ▸).

X-ray scattering measurements for CoO_*x*_-Bi thin films supported on planar 3 mm × 10 mm × 1.1 mm ITO glass electrode (Präzisions Glas & Optik GmbH) surfaces were measured at beamline 11-ID-D using a 23 keV X-ray beam vertically focused to 15 µm with a 0.3 m toroidal mirror. Different CoO_*x*_-Bi coated ITO electrodes were prepared having a CoO_*x*_-Bi layer thickness that varied from 0.5 µm to 2 µm (Kwon *et al.*, 2015 ▸) controlled by the electrolysis time. Following electrolysis, CoO_*x*_-Bi coated ITO glass electrodes were removed from the film-forming electrochemical cell, rinsed with distilled water and air dried. The ITO electrode surface was aligned parallel to the incident X-ray beam and positioned to traverse the 3 mm electrode dimension. Scattering patterns were measured on a Pilatus 2M detector (beamline 11-ID-D). Detector images were azimuthally averaged and corrected for angle-dependent X-ray absorption. For positioning the CoO_x_-Bi coated ITO electrode in the X-ray beam, the electrode assembly was vertically scanned in 5 µm steps while monitoring the X-ray scattering image. As the CoO_*x*_-Bi/ITO/glass electrode assembly progressed into the X-ray probe, position-dependent contributions from air, CoO_*x*_-Bi, ITO and glass could be discerned. A vertical position of the electrode was chosen to maximize the contribution from the amorphous CoO_*x*_-Bi layer compared with the crystalline ITO and glass support.

## 3. Results and discussion   

**Figure 3:**
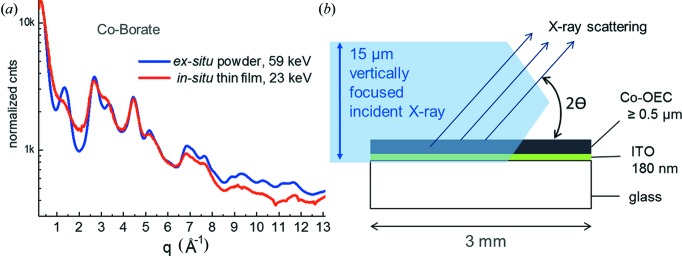
(*a*) Comparison of scattering patterns for the CoO_*x*_-Bi thin-film catalyst. The red line trace shows the scattering pattern measured as an ∼1 µm film supported on a planar ITO glass electrode in air using a 3 mm sample film path length with the grazing-incidence geometry sketched in part (*b*). The blue line trace shows a portion of the 59 keV scattering pattern measured for the CoO_*x*_-Bi powder sample in a 1 mm-diameter polyimide tube. (*b*) Grazing-incidence scattering geometry used for ITO-supported CoO_*x*_-Bi OEC thin-film measurements, using an ∼15 µm vertically focused 23 keV X-ray beam.

X-ray scattering for an ∼1 µm-thick CoO_*x*_-Bi film supported on a planar ITO glass electrode in air is shown in Fig. 3 ▸(*a*), interrogated using an ∼15 µm vertically focused 23 keV X-ray beam with grazing incidence parallel to the ITO glass surface, in which the maximum scattering angle, 2θ_max_, was 74° with a *q*_max_ of 13 Å^−1^. The scattering geometry is shown diagrammatically in Fig. 3 ▸(*b*). The parallel incidence scattering for the electrode-supported CoO_*x*_-Bi film in Fig. 3 ▸(*a*) is compared in this same *q*-range with a high-resolution HEXS pattern (59 keV) for a comparable CoO_x_-Bi film that had been scraped off the ITO surface and measured as an *ex situ* powder (Kwon *et al.*, 2015 ▸). Overall, the electrode-supported film measured by grazing-incidence and *ex situ* powder-scattering patterns showed good agreement in the *q*-range below 9 Å^−1^. Above this *q* value, deviations were seen that presumably reflect inhomogeneities arising from edge effects and distortions because of an acute angle of incidence of the scattered X-rays on the mosaic detector. We found that the grazing-incidence geometry was limited by two factors. First, the inability to extract high-quality scattering patterns for films with thicknesses below 0.5 µm because of the small fraction of the 15 µm focused X-ray beam that interacts with the thin film. This limitation was prohibitively severe when an electrolyte overlayer needed for *in situ* electrochemical measurements was added to the assembly. Second, the *q*_max_ attainable with a 23 keV beam restricts spatial resolution to about 0.48 Å. The thin-film limit could be addressed by the use of a sub-micrometre-focused X-ray beam, but the combination of a sub-micrometre vertically focused high-energy (>50 keV) X-ray beam would be needed to achieve high-spatial-resolution PDFs on interfacial 2D ultra-thin films. However, sub-micrometre-focused high-energy X-ray beams are not currently available at the APS.

**Figure 4:**
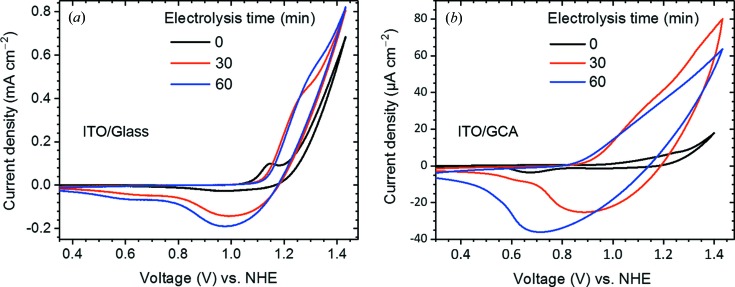
Comparison of cyclic voltammetric curves for planar ITO/glass (*a*), and ITO/Au/40 µm GCA (*b*) WEs in an electrolyte that contained 0.1 *M* phosphate, pH 7.0, and 0.5 m*M* Co(NO_3_)_2_·6H_2_O. CV traces were recorded using 5 mV s^−1^ scan rates, and following 0 min, 30 min and 60 min of otherwise continuous electrolysis at 1.34 V (versus NHE).

In order to enable PDF analyses of interfacial films with thickness below 0.5 µm and in the presence of electrolyte needed for *operando* measurements, we designed a microporous, high-surface-area electrode architecture that allows utilization of unfocused high-energy X-ray beams. We demonstrate this approach using a TCO-coated GCA. The electrochemical properties of an ITO/GCA assembly with 40 µm pores are shown in Fig. 4 ▸. Cyclic voltammograms (CVs) for the CoO_*x*_-Pi OEC on planar ITO/glass WE as a reference, Fig. 4 ▸(*a*), are compared with those measured for the ITO/GCA WE, Fig. 4 ▸(*b*). CVs were recorded at time points during continuous CoO_*x*_-Pi electrochemical deposition and film growth with 1.05 V (NHE) applied potential from an aqueous electrolyte solution containing 0.5 m*M* Co(NO_3_)_2_·6H_2_O and 0.1 *M* KPi, pH 7.0 (Kanan & Nocera, 2008 ▸; Surendranath *et al.*, 2009 ▸). On the first anodic sweep with a fresh ITO electrode, a peak is seen at 1.1 V (NHE) corresponding to the oxidation of the Co(II) solution and deposition of the Co(III) oxide, followed by a catalytic onset at 1.23 V (Kanan & Nocera, 2008 ▸; Surendranath *et al.*, 2009 ▸). With continuous electrolysis, the CoO_*x*_-Pi film thickens, and the anodic sweeps show plateau peaks that arise from the Co(+3/+2) redox couple and the insulator-to-semiconductor transition in the CoO_*x*_-Pi film (Costentin *et al.*, 2016 ▸; Surendranath *et al.*, 2010 ▸; Risch *et al.*, 2012 ▸, 2015 ▸; Klingan *et al.*, 2014 ▸; González-Flores *et al.*, 2015 ▸). Corresponding electrochemical features are seen in the CVs with the ITO/GCA WE, although the electrochemical transitions are broadened and slightly shifted, apparently because of increased series resistance and capacitance in the microporous electrode assembly. Comparable CVs were measured for both the ITO/GCA and IZO/GCA assemblies. Causes for the differences between planar and microporous TCO as WEs for CV measurements are not fully understood. Preliminary experiments suggest that the distortions in the CVs for the microporous compared with the planar TCO WEs cannot be recovered by increasing the ALD TCO layer thickness beyond the 50 nm–150 nm dimensions which are used for the *in situ* X-ray measurements described below. This suggests the possibility that contributions from electrochemically active surface sites may differ between the planar and microporous TCO electrode geometries. This aspect is being further investigated. At present, the TCO coatings described here are noted to allow adequate electrochemical control of the electrode and catalyst potential while also providing, as described below, an X-ray scattering background compatible with high-resolution *operando* PDF measurements.

**Figure 5:**
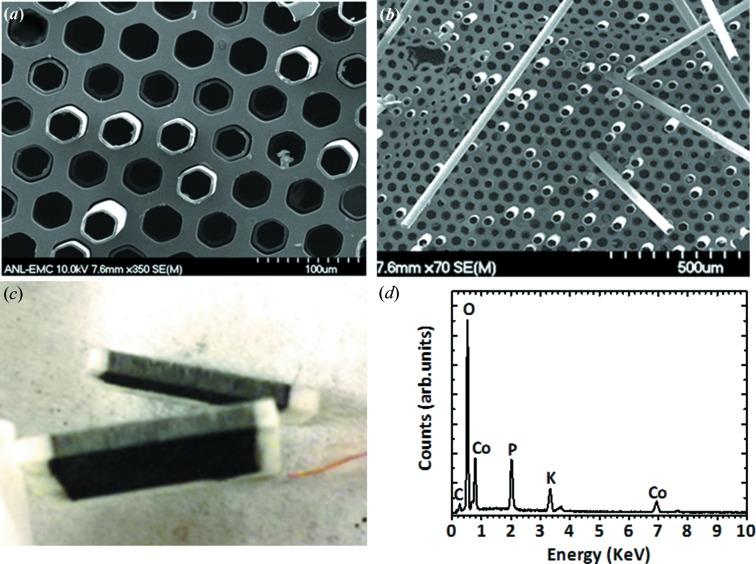
(*a*) SEM image of the electrodeposited CoO_*x*_-Pi catalyst film electrochemically deposited onto the ITO/GCA electrode support. (*b*) SEM expanded view showing uniform CoO_*x*_-Pi ‘tubes’ removed from the templating electrode pores. (*c*) Optical micrograph of the ITO/GCA electrode assembly supporting the electrochemically deposited CoO_*x*_-Pi thin-film catalyst, split in half, exposing the interior transverse pores. (*d*) EDX plot acquired at 12 kV. Carbon, oxygen, phosphorous and potassium originated from *K*α. Co at 6.929 keV and at 0.776 keV originated from *K*α and *K*β, respectively.

Figs. 5 ▸(*a*) and 5 ▸(*b*) show scanning electron micrographs of an ITO/GCA WE after a 4 h CoO_*x*_-Pi catalyst-film electrochemical deposition within the porous electrode. Upon drying, the CoO_*x*_-Pi film contracts allowing film to separate from the ITO pore surface. This results in the formation of the observed free-standing, pore-templated, tubular-shaped CoO_*x*_ films. The uniformity of tube-wall thickness along the length of each tube suggests that the ITO/GCA supports uniform electrochemistry throughout the length of each pore. The uniformity of the catalyst-film coating is also demonstrated by visual inspection of a cleaved ITO/GCA after electrolytic CoO_*x*_-Pi deposition, Fig. 5 ▸(*c*). Elemental composition analysis by energy-dispersive X-ray (EDX), Fig. 5 ▸(*d*), shows the composition and Co:P:K ratio of 3:1:0.7 of the catalyst film to be comparable with those measured from the CoO_*x*_-Pi catalyst deposited on planar ITO electrode surfaces (Kanan & Nocera, 2008 ▸). The uniformity of the electrochemical film deposition along the 1.1 mm GCA pore dimensions easily accommodates the cross section of an unfocused high-energy synchrotron beam, highlighting the opportunity to use these microporous supports for *in situ* PDF analysis of thin-film catalysts.

**Figure 6:**
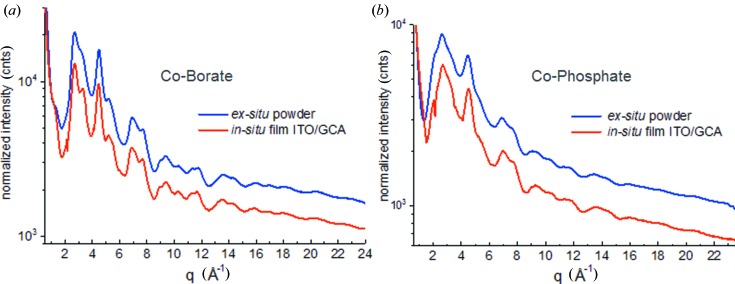
Comparison of HEXS (59 keV) patterns for (*a*) CoO_*x*_-Bi and (*b*) CoO_*x*_-Pi measured as ITO/GCA supported films (red traces) and as powder samples (blue traces) scraped from planar ITO electrodes and collected in 1 mm-diameter polyimide tubes.

To demonstrate this capability, HEXS patterns measured for CoO_*x*_-Bi and CoO_*x*_-Pi as *in situ* ITO/GCA supported films in the absence of electrolyte are shown in Figs. 6 ▸(*a*) and 6 ▸(*b*), respectively, and are compared with PDF patterns measured for corresponding samples that were electrolytically grown on planar ITO electrodes and scraped off and examined as *ex situ* powders (Du *et al.*, 2012 ▸; Kwon *et al.*, 2015 ▸; Farrow *et al.*, 2013 ▸). Powder samples removed from planar electrode surfaces currently serve as the samples of choice for high-resolution PDF measurements of transition metal oxide catalyst films. Fig. 6 ▸ reveals the equivalence of the HEXS patterns for powder and *in situ* films throughout the experimental *q*-range, demonstrating the capability to make high-resolution measurements on catalyst films *in situ*.

**Figure 7:**
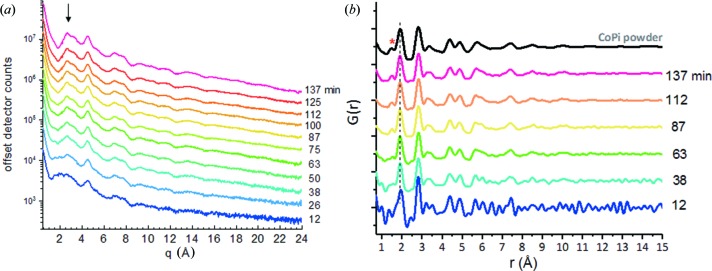
*In situ* X-ray monitoring of CoO_*x*_-Pi OEC during film growth on an ITO/GCA WE with 40 µm pores. (*a*) Background-subtracted [electrolyte-filled reference area as indicated in Fig. 2 ▸(*d*)] HEXS data. HEXS patterns were collected with 2 min integration times at selected time points during continuous electrolytic CoO_*x*_-Pi film deposition from an electrolyte solution containing 0.1 *M* potassium phosphate, pH 7.0, and 0.5 m*M* Co(NO_3_)_2_·6H_2_O with 1.85 V (versus NHE) applied potential. The X-ray beam was defined by 0.1 mm horizontal and 0.5 mm vertical slit apertures. (*b*) *G*(*r*) Fourier transforms from the HEXS data in (*a*). The top trace shows the *G*(*r*) of CoO_*x*_-Pi powder as a reference for comparison. Noise in the HEXS patterns was attenuated using a 24-point smoothing function in the program *PDFgetX2* (Qiu *et al.*, 2004 ▸) prior to Fourier transform. The smoothing only partially removes Fourier ripple artifacts in the ensuing PDF. The high-frequency noise in the reciprocal-space data converts to non-physical pair correlations below 0.7 Å (not shown). Longer acquisition times beyond the 2 min scans used in (*a*) would be needed to raise the signal-to-noise and avoid these artifacts for thin CoO_*x*_-Pi films with electrolysis times shorter than 38 min.

The ability to use the TCO/GCA WE for *operando* PDF analyses in an electrochemical cell is demonstrated in Fig. 7 ▸(*a*), which shows HEXS patterns measured with 2 min acquisition times sampled at various points during the time course of continuous electrolysis and CoO_*x*_-Pi film deposition at 1.85 V versus NHE. We note that compared with the HEXS measurements for the ITO/GCA supported films in air, Fig. 5 ▸, the *operando* measurements shown in Fig. 7 ▸ include the presence of flowing electrolyte in the GCA pores. An additional benefit of the microporous GCA with approximate 64% porosity is that it restricts the electrolyte contribution to background scattering. TCO/GCA background-subtracted HEXS, Fig. 7 ▸(*a*), and corresponding PDF patterns, Fig. 7 ▸(*b*), demonstrate the opportunity to utilize the TCO/GCA to track real-time deposition, growth and structural evolution of amorphous CoO_*x*_-Pi catalyst thin films. The HEXS and PDF patterns for supported films derived from the *operando* HEXS data are quite similar to those of the *ex situ* powder samples (Kwon *et al.*, 2015 ▸), although there is evidence for time evolution in the fine features of these scattering signals. Interestingly, the position of the PDF peak at 1.95 Å, arising from the first coordination shell Co–O distance (Du *et al.*, 2012 ▸), is shifted ∼0.04 Å toward a longer radial distance in the early 12 min time point compared with later traces, as seen in Fig. 7 ▸(*b*). This bond-distance increase is consistent with the presence of lower-valence cobalt in the thinnest films, which is more like the Co^2+^ species in the electrodeposition solution. The 12 min HEXS pattern shows a broadening of low-*q* features compared with later traces, marked by the arrow in Fig. 7 ▸(*a*). The PDF in Fig. 7 ▸(*b*) also suggests structural evolution in the detection of the phosphorous–oxygen bond distance peak at 1.50 Å (marked by the asterisk) over time. The signal-to-noise in the 12 min time-point trace is limited by the single image, 2 min acquisition time. Improvements in signal-to-noise for a thin-film sample can be achieved by taking averages of the repeated single time point or by extending the integration time.

**Figure 8:**
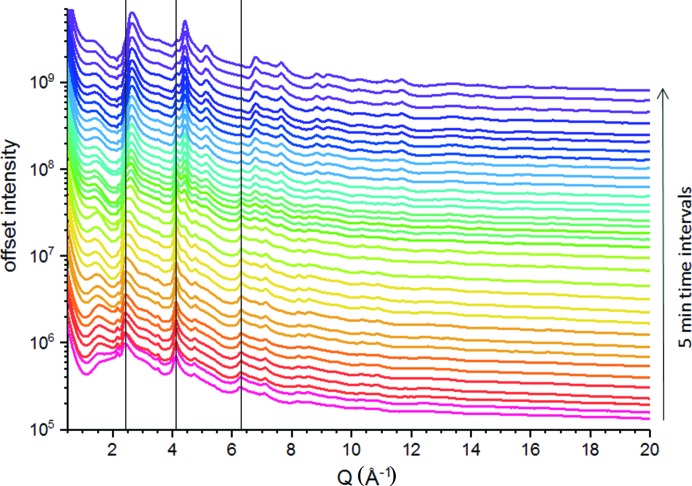
*In situ* X-ray monitoring of CoO_*x*_-Bi OEC during film growth on an ITO/GCA WE with 40 µm pores. The traces show background-subtracted [electrolyte-filled reference area as indicated in Fig. 2 ▸(*d*)] HEXS patterns collected with 4.5 min integration at 5 min time intervals during continuous electrolytic CoO_x_-Bi film deposition from an electrolyte solution containing 0.1 *M* potassium borate, pH 9.2, and 0.5 m*M* Co(NO_3_)_2_·6H_2_O with 1.4 V (versus NHE) applied potential. For reference, the vertical lines mark the positions of the scattering peaks at *q* = 2.43 Å^−1^, *q* = 4.10 Å^−1^ and *q* = 6.31 Å^−1^ as seen in the first HEXS pattern. HEXS patterns were shifted vertically by multiplication for clarity. The X-ray beam was defined by 0.1 mm horizontal and 0.5 mm vertical slit apertures.

For comparison, Fig. 8 ▸ shows difference HEXS patterns (CoO_*x*_-Bi/ITO/GCA − ITO/GCA) measured with 4.5 min integration time recorded at 5 min intervals during an analogous CoO_*x*_-Bi OEC film deposition with continuous electrolysis from a circulating electrolyte solution that contained 0.1 *M* potassium borate, pH 9.2, and 0.5 m*M* Co(NO_3_)_2_·6H_2_O with 1.4 V (NHE) applied potential. The initially deposited film shows a pattern of scattering peaks, for example marked at *q* = 2.43 Å^−1^, 4.10 Å^−1^ and 6.31 Å^−1^, on an amorphous scattering background. During CoO_*x*_-Bi film growth, the scattering patterns show continuous progression that reflect a characteristic evolution in CoO_*x*_-Bi OEC film structure. A detailed analysis of time-dependent changes in CoO_*x*_-Pi and CoO_*x*_-Bi OEC film structures during electrochemical deposition will be presented elsewhere. Here we highlight these results to demonstrate the TCO/CGA as a WE design that has general applicability for *operando* structural characterization of first-row transition-metal thin films using HEXS and PDF analyses. The high surface area of these electrodes compensates for the low-scattering cross-sections with high-energy X-rays.

**Figure 9:**
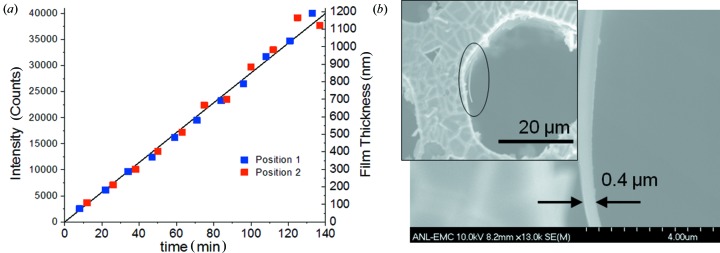
Time-dependent *in situ* measurement of CoO_*x*_-Pi electrochemical film deposition. (*a*) Plot of HEXS signal intensity at selected times during the continuous electrochemical deposition, as described in Fig. 7 ▸. The scattering intensity was measured by the amplitude of the peak feature at *q* = 4.5 Å^−1^ and recorded for two positions along the ITO/GCA pore. The first position was placed just below the top edge of the ITO/GCA, the second 0.1 mm below this spot, shown as blue and red symbols, respectively. The X-ray beam was defined by 0.1 mm horizontal and 0.5 mm vertical slit apertures. (*b*) SEM image of CoO_*x*_-Pi film on the ITO/GCA electrode following 50 min of electrolysis, using the conditions described in Fig. 7 ▸. The inset shows a field-of-view imaging of one of the ITO/GCA pores. The circle marks the area of enlargement shown on the right.

An assessment of sensitivity for *operando* HEXS measurements was made by comparison of HEXS amplitudes to film-thickness estimates made from scanning electron microscopy (SEM), Fig. 9 ▸. Fig. 9 ▸(*a*) shows a plot of scattering intensities measured for the peak of the CoO_*x*_-Pi at *q* = 4.5 Å^−1^ as a function of film-deposition time, using the time-sequence dataset shown in Fig. 7 ▸(*a*). The data were collected at two positions along the pore channels. The first position was approximately 50 µm below the top surface of the TCO/GCA assembly, the second shifted 100 µm along the pore direction below this. The results show the linearity of film electrodeposition with time, and the uniformity of film deposition along the pore axis. In a separate equivalent ITO/GCA electrode, a parallel CoO_*x*_-Pi electrochemical deposition was run for 50 min, stopped, rinsed with water, dried and used for SEM analysis. Fig. 9 ▸(*b*) shows the resulting SEM images for this electrode. Portions of the CoO_*x*_-Pi film are seen to detach from the pore wall upon drying in vacuum. From these spots, the thickness of the CoO_*x*_-Pi film can be seen in cross section, and measured to be about 400 nm-thick after 50 min of electrolysis. This permits an approximate film-thickness scaling, as shown by the right-hand axis in the time-dependent *in situ* CoO_*x*_-Pi deposition in Fig. 9 ▸(*a*). The calibration shows that the 40 µm pore TCO/GCA provides a sensitivity sufficient to detect HEXS for amorphous CoO_*x*_-Pi films with 60 nm thickness.

The film-thickness sensitivity in these *operando* experiments is primarily set by the volume fractions of the electrolyte and the GCA support within the TCO/GCA assembly. The TCO layer makes only a small volume contribution. For example, for the ITO/GCA assembly used in Figs. 7 ▸–9 ▸, the GCA was composed of hexagonally packed, 40 µm diameter, rounded hexagonal-shaped pores with a 50 µm center-to-center pore spacing. From this we estimate the volume fractions of the electrolyte-filled pore, GCA support, 40 nm ITO coating and 60 nm CoO_*x*_-Pi film within the WE assembly to be 0.633, 0.360, 0.003 and 0.004, respectively. Adjustments to the TCO layer thickness in the 40 nm to 150 nm range cause only a small variation in the volume contribution because of the CoO_*x*_ film, and in all cases the limiting 60 nm film thickness can be estimated to have an ∼0.4% volume contribution under *operando* conditions. The experimental data are consistent with this estimate. For example, the ITO/GCA subtracted scattering signal from the CoO_*x*_-Pi supported film WE at the 12 min time point in Figs. 7 ▸ and 9 ▸(*a*) had 3236 detector counts during the 2 min acquisition time measured for the peak at *q* = 4.5 Å^−1^, which was 0.33% of the total scattering signal. At higher angle, *q* = 20 Å^−1^, the CoO_*x*_-Pi film scattering signal diminished to 421 counts, representing 0.1% of the total *operando* WE scattering. Our experiments with ALD coatings with GCAs having different diameters show that HEXS signals for thin films scale with the porous electrode surface area. Based on these considerations, we anticipate that a strategy for accomplishing PDF analyses of sub-nanometre supported films could be achieved by diminishing the volume contributions of the pore-filled electrolyte and porous support. We note that porous anodic alumina oxide (AAO) membranes offer one such assembly (Elam *et al.*, 2003 ▸, 2006 ▸). For example, we estimate that an AAO membrane with 50% porosity and 20 nm TCO-coated 200 nm pores would provide a WE with over 100-fold higher surface area than the 40 µm ITO/GCA assemblies used here, and offer the opportunity to extend the PDF analyses of sub-nanometre films.

**Figure 10:**
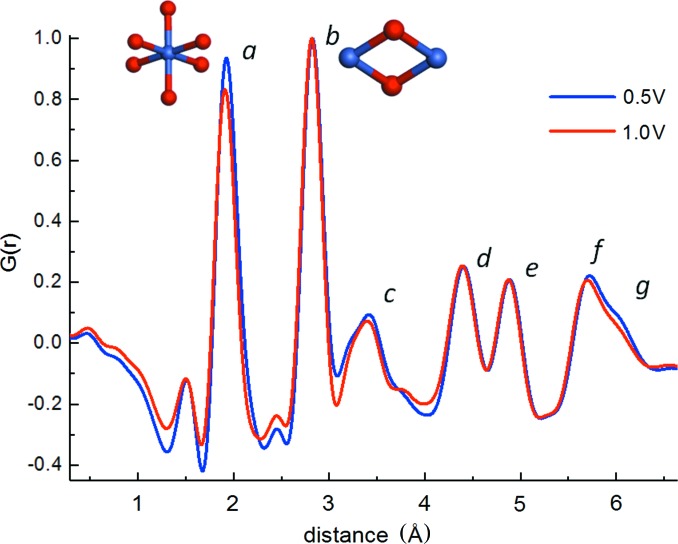
*Operando* PDF analysis for the CoO_*x*_-Pi OEC, resolving the change in fine structure associated with redox state cycling between 0.5 and 1.34 V (versus NHE). A CoO_*x*_-Pi OEC was grown on an IZO/GCA WE by 60 min electrolysis at 1.34 V (versus NHE) in 0.1 *M* phosphate, pH 7.0, and 0.5 m*M* Co(NO_3_)_2_·6H_2_O. Subsequently, HEXS and PDF patterns were measured as a function of applied potential that was cycled between 0.5 V, 1.0 V and 1.5 V (versus NHE). The figure compares PDF patterns measured at 0.5 V and 1.0 V in the blue and red line traces, respectively. The peaks labeled *a* through to *g* correspond to atom-pair distances that are characteristic of the cobaltate domain structure (Du *et al.*, 2012 ▸; Kwon *et al.*, 2015 ▸). For example, the peak *a* is associated with the first-shell Co–O ligand distances, and peak *b* corresponds to the Co–Co distance across the di-μ-oxo bond. Illustrative structures are inserted next to each of these peaks. The cobalt and oxygen atoms are colored blue and red, respectively.

Finally, we found the TCO/GCA to be a suitable WE for *in situ* electrochemical fine-structure analysis by *operando* HEXS. For example, Fig. 10 ▸ shows PDF patterns measured for a CoO_x_-Pi OEC film that was cycled between electrochemical potentials of 0.5 V, 1.0 V and 1.5 V (versus NHE), corresponding to electrochemical regimes where the di-μ-oxo-linked cobalt pairs are considered to be in the Co(+3/+2), Co(+3/+3) and Co[(mixture: +3, +4)/+3] oxidation states, respectively (Costentin *et al.*, 2016 ▸; Surendranath *et al.*, 2010 ▸; Risch *et al.*, 2012 ▸, 2015 ▸; Klingan *et al.*, 2014 ▸; González-Flores *et al.*, 2015 ▸). The CoO_*x*_-Pi was first grown on an IZO/GCA electrode for 60 min by electrolysis at 1.34 V (versus NHE) in 0.1 *M* phosphate, pH 7.0, and 0.5 m*M* Co(NO_3_)_2_·6H_2_O. Fig. 10 ▸ compares PDF patterns measured at 0.5 V and 1.0 V. The PDF patterns correspond to the 13 cobaltate atom domain model described previously, in which each of the PDF peaks can be correlated with atom-pair distances in the domain model (Du *et al.*, 2012 ▸; Farrow *et al.*, 2013 ▸; Kwon *et al.*, 2015 ▸). Peaks labeled *a*, *c* and *g* correspond to Co–O atom pairs that include terminal oxygen atoms at the domain edge. The shortest Co–O peak, *a*, arises from the first-shell ligand bonding distance. Peaks *b*, *d*, *e* and *f* correspond to Co–Co atom pairs, with peak *b* arising from the distance between di-μ-oxo-linked cobalt atoms (Du *et al.*, 2012 ▸; Farrow *et al.*, 2013 ▸; Kwon *et al.*, 2015 ▸). Starting from a potential of 0.5 V, an increase in the oxidation potential to 1 V is accompanied by a slight atom-pair distance shortening, seen in a 0.02 Å shift in the position of peak *a*, which is consistent with the oxidation of some of the cobalt centers in the domain and is comparable with results from extended XAFS measurements (Risch *et al.*, 2015 ▸). Detectable too are slight changes to the amplitudes of peaks *a*, *c* and *g* that include Co–O atom pairs involving terminal oxygen atoms. In contrast, the peaks *b*, *d*, *e* and *f* associated with Co–Co atom pairs in the domain lattice show no amplitude alteration. We interpret these results as supporting a model in which redox activity is localized at the domain edges, and is associated with a disordering of terminal oxo coordination geometries. The PDF fine-structure changes are seen to be reproducible upon cycling between 0.5 V, 1.0 V and 1.5 V. An increase in potential from 1.0 V to 1.5 V produces no additional changes in PDF features. A more complete analysis and discussion of redox-state-dependent fine-structure changes in Co-oxide OEC thin films will be presented and discussed elsewhere. However, here we use these results to demonstrate the opportunity to use the TCO/GCA as a robust, high-surface-area WE for electrochemical-based PDF structure analyses of catalytic thin films by HEXS.

## 4. Conclusion   

*Operando* PDF analysis via HEXS of high-surface-area electrodes enables the first opportunity to detect structure and structural evolution during the growth of electrochemically deposited films. The new experimental design further affords the opportunity to resolve structure as a function of an applied potential under water-oxidation conditions for catalytic thin films with thicknesses that range from 60 nm to micrometre scales. The TCO/GCA electrode design successfully addresses three technical challenges for high-sensitivity HEXS measurements. Specifically, the design: (i) provides a high surface area that is needed to compensate for low X-ray scattering cross-section by high-energy X-rays when interrogating thin films composed of low-*Z* atoms, (ii) limits the electrolyte volume and therefore the contribution to background scattering, and (iii) delivers an amorphous silica template and nanoscale TCO coating by ALD with minimal HEXS background. A custom 3D-printed electrochemical cell was shown to allow precise and reproducible arrangement of the three-electrode setup. Finally, the results demonstrate the opportunity to combine advances in high-surface-area porous materials and ALD to produce tunable substrates for interfacial PDF analyses. The scalability of the interfacial PDF measurement to pore size and surface area suggests the opportunity to extend the interfacial catalyst analysis to the molecular scale by using porous supports with sub-micrometre pore dimensions.
